# High-Performance Four-Channel Tactile Sensor for Measuring the Magnitude and Orientation of Forces

**DOI:** 10.3390/s24092808

**Published:** 2024-04-28

**Authors:** Mingyao Zhang, Yong Shi, Haitao Ge, Guopeng Sun, Zihan Lian, Yifei Lu

**Affiliations:** School of Mechanical Engineering, Heilongjiang University, Harbin 150001, China; 2221851@s.hlju.edu.cn (M.Z.); 2222791@s.hlju.edu.cn (H.G.); 2232789@s.hlju.edu.cn (G.S.); 2232777@s.hlju.edu.cn (Z.L.); 2221842@s.hlju.edu.cn (Y.L.)

**Keywords:** flexible sensor, tactile sensor, multi-channel mechanism, linear response, under-actuated robotic hand

## Abstract

Flexible sensors have gained popularity in recent years. This study proposes a novel structure of a resistive four-channel tactile sensor capable of distinguishing the magnitude and direction of normal forces acting on its sensing surface. The sensor uses Ecoflex^TM^00-30 as the substrate and EGaIn alloy as the conductive filler, featuring four mutually perpendicular and curved channels to enhance the sensor’s dynamic responsiveness. Experiments and simulations show that the sensor has a large dynamic range (31.25–100 mΩ), high precision (deviation of repeated pressing below 0.1%), linearity (R2 above 0.97), fast response/recovery time (0.2 s/0.15 s), and robust stability (with fluctuations below 0.9%). This work uses an underactuated robotic hand equipped with a four-channel tactile sensor to grasp various objects. The sensor data collected effectively predicts the shapes of the objects grasped. Furthermore, the four-channel tactile sensor proposed in this work may be employed in smart wearables, medical diagnostics, and other industries.

## 1. Introduction

Skin, as a crucial organ for sensing the external environment, provides the brain with information about the material and shape of objects it touches. Flexible pressure sensors can detect external pressure information, and thus convert mechanical stimuli into electrical signals. Nowadays, flexible sensors have various applications, including wearable electronic skin [1,2], smart wearables [3], and medical health monitoring [4,5]. Pressure sensors can be categorized into four groups based on their functionality: resistive [6,7,8], capacitive [9,10], piezoelectric [11,12], and friction-based [13]. While capacitive sensors offer high sensitivity and a wide measurement range, they are susceptible to electromagnetic interference [14]. Friction-based sensors, despite their low power consumption, may not be suitable for all tactile tasks [15]. Piezoelectric sensors exhibit high dynamic properties and stability, but their sensitivity to inertial loads can lead to poor signal stability [16,17]. Resistive sensors, on the other hand, have the advantages of high linearity and sensitivity, ease of fabrication and low cost, which can efficiently convert external mechanical stimuli into resistance changes [18,19,20]. Consequently, they are widely used in robotic skin and smart wearables.

The selection of substrate materials and internal conductive fillers is crucial for the performance of flexible sensors. PDMS [21,22], Ecoflex [23], and DragonSkin [24] are frequently utilized as flexible sensor substrates for their exceptional stretchability, stability, and biocompatibility. Among these materials, Ecoflex stands out because of its superior water resistance, rip resistance, lower Young’s modulus, greater stretchability, and improved biocompatibility [25]. Carbon black (CB) [26], EGaIn alloy [27], carbon nanotubes (CNTs), graphene, and ionic liquids have attracted much attention in the study of conductive fillers for flexible sensors. Compared with other materials, EGaIn alloys exhibit superior material properties, such as strong fluidity and high surface tension at room temperature, low viscosity (1.98 × 10^−3^ Pa·s), and excellent conductivity [28]. These properties allow the alloy to deform well with flexible sensor channels.

In recent years, significant progress has been made in the field of micro-nano engineering. By combining elastic substrates, such as polydimethylsiloxane, polyurethane, and Ecoflex, with active nanomaterials, like carbon nanotubes, gold, silver, micronanowires, and MXene, more conductive pathways can be formed under external pressure, resulting in flexible tactile sensors with enhanced piezoresistive sensitivity [29,30]. However, sensors fabricated by micro-nanotechnology are prone to malfunction due to issues with complicated design, cumbersome fabrication procedures, and challenging process control. For example, anisotropic structures prepared with femtosecond lasers exhibit significant fluctuations in readings, even under minor forces [31]. To solve this problem, pyramids, hemispheres, cylinders [32], fiber [33], and other structures are incorporated into tactile sensors to expand the contact area per unit area, and thus enhance the response speed of sensors. However, the anisotropic nature of sensors often leads to premature saturation of sensor linearity and piezoresistive behavior [34]. Furthermore, single-channel tactile sensors can only provide information about the normal force received by the contact surface without being able to determine the specific direction of force applied to the surface [35]. Therefore, it is essential to develop a sensor capable of recognizing normal stress and its direction, while also exhibiting good linearity and high responsiveness.

In recent years, the application of sensors in smart robotic hands has gained widespread attention. Reference [36] describes a technology using 3D-printed PVA stents and hydrogel casting to manufacture an exoskeleton hand capable of accurately distinguishing music performance. Another study [37] equips a robotic hand with tactile sensors to provide tactile feedback, offering amputees the possibility of possessing highly dexterous prosthetic limbs. The installation of stretchable sensors on the back of an underactuated robotic hand allows the shape of an object being grasped to be analyzed based on the bending angles of the joints [38]. Therefore, it is crucial to provide robotic hands with tactile feedback akin to human hands.

This study focuses on the development of an affordable and flexible four-channel piezoresistive tactile sensor. Unlike traditional tactile sensors, this novel sensor enhances the ability to determine force direction and recognize shapes. By mimicking the human skin’s ability to sense external pressure, it allows underactuated robotic hands to effectively identify the shapes of objects. The four-channel tactile sensor creates new opportunities and solutions in the field of human–machine interaction.

## 2. Four-Channel Tactile Sensor

### 2.1. The Structural Design and Fabrication of Tactile Sensors

**Figure 1:**
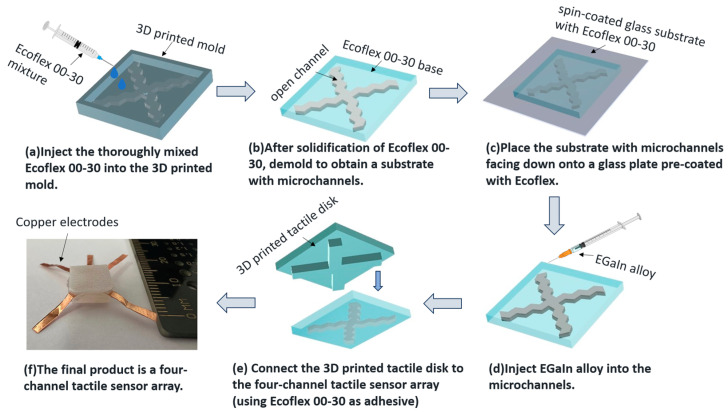
Schematic of the fabrication process for the four-channel tactile sensor.

The fabrication for the four-channel tactile sensor is depicted in Figure 1. First, the Ecoflex^TM^00-30 A and B components are mixed in a 1:1 ratio for five minutes. Then, pour the mixed Ecoflex^TM^00-30 into a plastic mold containing pre-attached copper electrodes (Figure 1a). The entire structure is baked in an oven at 80 °C for one hour. After curing, the silicone base will form an open channel 1 mm wide, 1 mm high, and 6 mm long (Figure 1b). A thin layer of Ecoflex^TM^00-30 (less than 200 μm thick) is spin-coated onto the glass substrate to form an enclosed channel, which is then wet-bonded to the silicone base with the open channel to form a closed channel (Figure 1c). The EGaIn alloy is injected into the channel using a micro-syringe, while air is evacuated from the opposite end using a separate syringe, as shown in Figure 1d. Finally, after the EGaIn is filled into the channel, the tactile sensor is encapsulated with Ecoflex^TM^00-30 spin coating. The fabricated sensor measures 1 cm × 1 cm in size, and is less than 2 mm thick.

Use a 3D printer to create a tactile disk containing four rectangular touch points. This tactile disk will be placed over the top of a flexible sensor. Use Ecoflex^TM^00-30 as an adhesive to bond the tactile disk with the four-channel tactile sensor (Figure 1e). Finally, spin-coat EcoflexTM00-30 on each surface of the tactile sensor. After curing, the encapsulated tactile sensor is obtained, as shown in Figure 1f. The final size of the sensor is 1 cm × 1 cm, with a height of 3.5 mm.

### 2.2. The Principle and Testing of Individual Channels

#### 2.2.1. The Operating Principle of an Individual Channel

**Figure 2:**
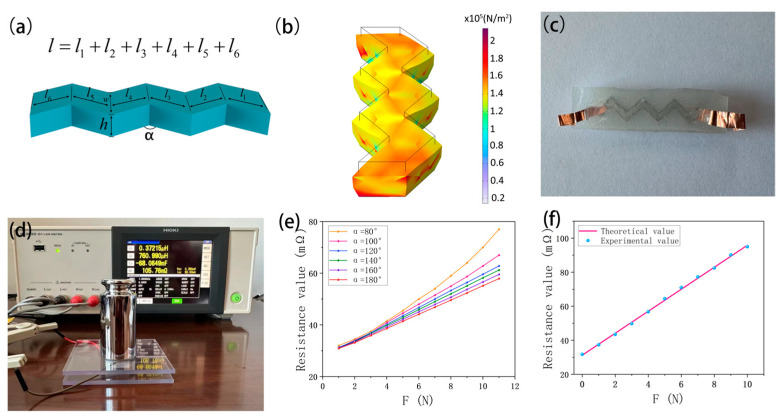
(**a**) Schematic of the structural parameters of the four-channel tactile sensor. (**b**) Deformation generated by the compressed simulation channel. (**c**) Physical representation of a single channel of the four-channel tactile sensor. (**d**) The experimental environment of the four-channel tactile sensor. (**e**) Scatter plot of experimental data corresponding to six different α values of the sensor. (**f**) Distribution of 10 sampled data points on the theoretical data line.

The single-channel structure adopts a zigzag pattern, as shown in Figure 2a. Figure 2b illustrates a finite element simulation, with 100 kPa pressure acted above the sensor. Despite the deformation of the channels, the compressed channel maintains its shape well, due to the surface tension effect of the EGaIn alloy. The vertical pressure causes an increase in channel width (an increase of 27.9%, relative to the initial width) and a decrease in height (a decrease of 38.7%, relative to the initial height). The change rate of channel length due to compression is less than 3.7%, with minimal impact on resistance values, and is thus negligible. The ideal resistance *R* of each channel of the four-channel tactile sensor under compression is deduced as follows:

(1) $$∆w=V_{Ecoflex}\cdot \frac{F}{A\cdot E_{Ecoflex}}$$

(2) $$∆h=-\frac{F}{A\cdot E_{Ecoflex}}$$

(3) $$R_{0}=\rho_{metal}\cdot \frac{l}{w\cdot h}$$

(4) $$∆R=R_{0}\cdot \frac{∆w}{W}-\frac{∆h}{h}$$

(5) $$R=R_{0}+∆R=R_{0}(1+V_{Ecoflex}\cdot \frac{F}{A\cdot E_{Ecoflex\cdot w}}+\frac{F}{A\cdot E_{Ecoflex\cdot h}})$$

where w, h and l represent the width, height, and length of the channel, respectively, $E_{Ecoflex}$ is the Young’s modulus of Ecoflex^TM^00-30 (750 [kPa]), $V_{Ecoflex}$ is the Poisson’s ratio of Ecoflex^TM^00-30 (0.49), $\rho_{metal}$ is the resistivity of the EGaIn alloy (2.5 × 10^−5^ [Ω/m]), A is the area of the force applied to the top of the channel, F is the magnitude of the applied force, $R_{0}$ is the initial resistance of the four-channel tactile sensor, and ∆R is the change in resistance due to deformation.

According to the theoretical model, the rate of change in resistance is related to the length, width, and height of the channel when the base area of a single touch point on the tactile disk is fixed. Although reducing the width and height of the channel can enhance sensor sensitivity, it will increase the difficulty and cost of manufacturing the sensor. Therefore, increasing the channel length, i.e., reducing the angle α of channel curvature, can improve sensor sensitivity, while maintaining a similar structure.

#### 2.2.2. Single-Channel Testing and Results

(1) The impact of channel curvature α on linearity

Place single-channel sensor samples with different bending angles α (shown in Figure 2c) on the test bench. Place weights of varying masses on the sensors, and then use an LCR meter (IM3533-01) to measure the resistance values of the sensors (as shown in Figure 2d). Figure 2e shows the resistance values of the sensor at bending angles α of 180°, 160°, 140°, 120°, 100°, and 80°. As the α decreases, the sensor’s sensitivity increases. However, as α continues to decrease, the relationship between stress and resistance becomes increasingly nonlinear. This is consistent with findings in reference [34], which suggest that increasing the contact area to enhance sensitivity leads to nonlinear sensor readings. When α is greater than 120°, the sensor readings maintain good linearity. Therefore, this study proposes a bending angle α of 120° for the channels of the four-channel tactile sensor for optimal performance.

(2) Linearity test

The linearity test was conducted on a single channel of a tactile sensor: a total of 10 sampling points were taken during a compression test (loads varying from 0 N to 10 N). Figure 2f illustrates the distribution of sampled values alongside the theoretical value line. The maximum error between experimental data and theoretical values is less than 0.34%, demonstrating excellent linearity in sensor readings.

(3) Performance test

**Figure 3:**
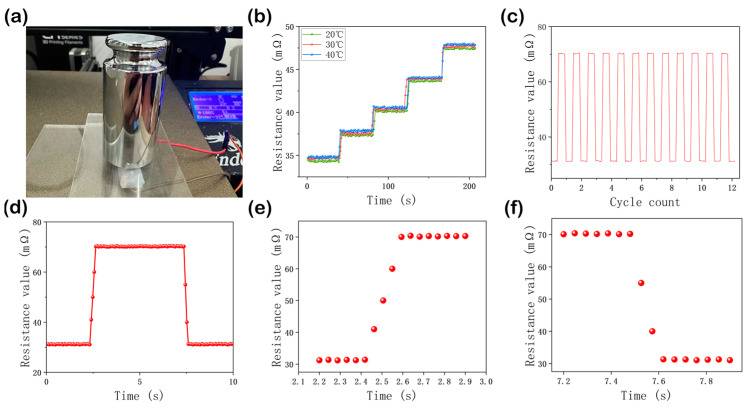
(**a**) illustrates the compression experiments conducted with the tactile sensor placed on a heating stage. (**b**) depicts the resistance changes in a single channel under the influence of multiple pressures at different temperatures. (**c**) shows the impact of continuous repetitive pressing on the readouts of a single channel of the sensor. (**d**) tracks the fluctuations in the sensor’s readings over a 10-s cycle. (**e**,**f**) display the rise and fall of the sensor’s readings within a cycle, reflecting the sensor’s response time (0.2 s) and recovery time (0.15 s).

To evaluate the performance of the sensor under different temperature and stress conditions, the sensor was tested on a heating stage (Figure 3a). Weights ranging from 50 g to 250 g were applied to a single channel at 20 °C, 30 °C, and 40 °C, and the sensor’s resistance values were recorded. The results indicate that the four-channel tactile sensor exhibits minimal temperature interference (fluctuations less than 0.01%) and excellent stability (fluctuations under equal pressure less than 0.4%) in room temperature conditions (20–40°C), as shown in Figure 3b. A single channel of the tactile sensor underwent 1000 compression tests with stress ranging from 0% (2 N) to 70% (6 N). Figure 3c displays the results of repeated stress over 12 cycles, where a 600 g weight was applied every 5 s. It demonstrates a high degree of stability, with deviations of peaks from troughs of less than 0.1%. Figure 3d shows the fluctuations within one cycle. Figure 3e and f respectively illustrate the areas where the number of indications rises and falls, reflecting the response time (0.2 s) and recovery time (0.15 s) of the sensor.

### 2.3. The Working Principle and Testing of the Four-Channel Tactile Sensor

#### 2.3.1. The Force Calculation Model of the Sensor

**Figure 4:**
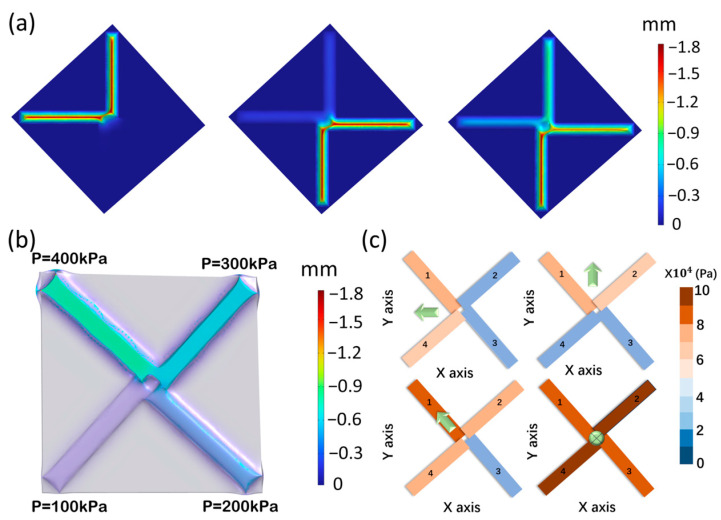
(**a**) Stress distribution of the four channels of the tactile sensor under compression. (**b**) Schematic representation of the performance of the tactile sensor under spatial forces. (**c**) Deformation of the units of the four-channel tactile sensor under different stresses, with arrows indicating the direction of stress.

Since four touch points of the four-channel tactile sensor are evenly distributed within 360°, they can be understood as components in four directions on a plane coordinate system. Let channel 2 be in the positive direction of the X-axis and channel 1 be in the positive direction of the Y-axis, as shown in Figure 4c. Due to the good linearity of a single channel of the tactile sensor, the magnitude and direction of the force on the tactile disk can be determined from the resistance values of the four channels.

(6) $$\theta =atan2(R_{Y},R_{X})$$

(7) $$F_{sum}=\sum_{i=1}^{4}\left(\frac{\left(R_{i}-R_{i0}\right)\cdot A\cdot E_{Ecoflex}\cdot w\cdot h}{R_{i0}\left(V_{Ecoflex}+w\right)}\right)(i=1,2,3,4)$$

As indicated in Figure 4c for channel numbering, $R_{X}$ denotes the force component of $F_{sum}$ along the X-axis ($R_{1}-R_{3}$), $R_{Y}$ the force component of $F_{sum}$ along the Y-axis ($R_{2}-R_{4}$), and θ is the angle between the resultant force vector and the X-axis.

#### 2.3.2. Testing of the Four-Channel Tactile Sensor

(1) Simulat

The relationship between stress and deformation in pressure sensors is simulated using COMSOL Multiphysics 6.0 software. The parameters of the model are 10 mm × 10 mm × 2 mm. It features a single zigzag channel inside, with a length of 6 mm, a width of 1 mm, and a height of 1 mm. Each channel has a stressed surface above it, measuring 4.8 mm in length and 2 mm in width. Apply pressure at different positions above the tactile disk of the four-channel tactile sensor. Each channel of the tactile sensor will experience various deformations, as shown in Figure 4a. Figure 4b displays the deformation of the contact surfaces when the four channels of the tactile sensor receive pressures of 400 Pa, 300 Pa, 200 Pa, and 100 Pa, respectively. The results show that each channel produces a different resistance response when the sensor is subjected to forces from different directions. By measuring the resistance of each channel after force application, touch can be fully sensed, and the size and direction of the force can be distinguished. The direction and quadrant of the load under different conditions can be obtained by analyzing the stress from the four channels of the sensor, as illustrated in Figure 4c.

(2) Experiment and Results

**Figure 5:**
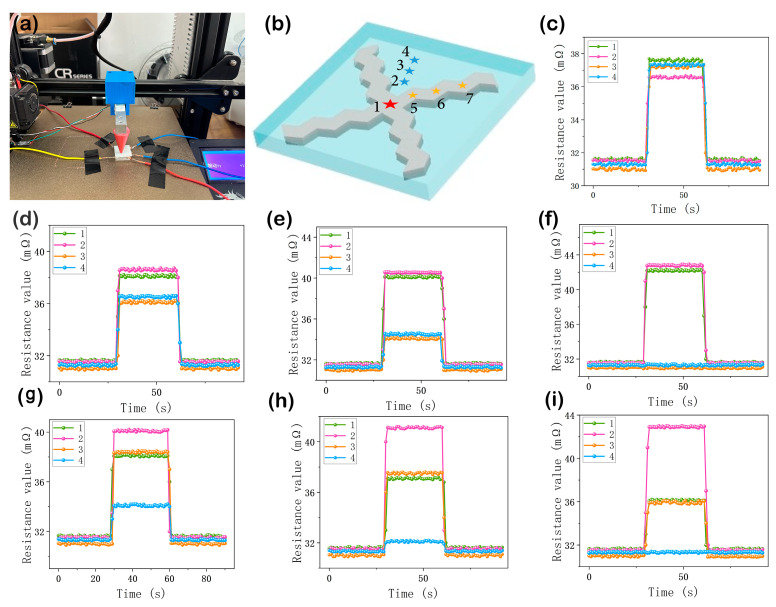
(**a**) Experimental setup. (**b**) Schematic diagram of experimental points. (**c**) Variation in resistance values of the four channels when a force of 3.5 N is applied at the center of the four-channel tactile sensor. (**d**–**f**) Variation in resistance values of the four channels when a force of 3.5 N is applied at points 2, 3, and 4 of the four-channel tactile sensor, respectively. (**g**–**i**) Variation in resistance values of the four channels when a force of 3.5 N is applied at points 5, 6, and 7, respectively.

The four-channel tactile sensor was subjected to a pressure test, as shown in Figure 5a. The load applied to the tactile sensor can be categorized into three scenarios: at the center of the sensor, on a single channel of the sensor, and between two channels of the sensor. Therefore, a total of seven experimental points were set up in this experiment, as illustrated in Figure 5b. A load of 3.5 N was applied at each experimental point. Between channels 1 and 2, experimental points 2, 3, and 4 were established at intervals of 0.6 mm along the main diagonal, and on channel 1, experimental points 5, 6, and 7 were set at intervals of 0.6 mm. The data for experimental point 1 are shown in Figure 5c, indicating a similar magnitude of resistance variation across all four channels. Data for experimental points 2, 3, and 4 are shown in Figure 5d, Figure 5e, and Figure 5f, respectively, while data for experimental points 5, 6, and 7 are shown in Figure 5g, Figure 5h, and Figure 5i, respectively.

The experimental data are shown in Table 1. The error between the calculated force ($F_{sum}$) and the actual loads (3.5 N) is less than 0.3%, and the deviation between the resultant force direction (θ) and the actual angle is below 4%. Both simulations and experiments verify that the four-channel tactile sensor proposed in this paper can discern the magnitude and direction of the normal force acting on the sensor.

It is noteworthy that each channel of the sensor employs a 120° zigzag configuration, representing a distinctive design aimed at not only enhancing the sensitivity but also maximizing the dispersion of forces generated by compression within a single channel, thereby ensuring that the EGaIn alloy predominantly remains within the channel. Although interconnected common electrodes may permit a minute amount of the liquid metal to migrate to adjacent channels, leading to slight fluctuations in readings, these minor fluctuations do not compromise the overall performance of the sensor. As demonstrated in the compression tests shown in Figure 5, the resulting errors from these fluctuations are negligible and do not adversely affect the sensor’s functionality.

## 3. Underactuated Robotic Hand for Wearable Applications

This work involves grasping experiments by using an underactuated robotic hand, which is composed of four structurally identical fingers (each with three joints), a thumb (with two joints), and a relatively compliant palm. The fingers are operated by internal tendon wires and connected by polyurethane rubber tendons between each joint, so the fingers are able to automatically return to their original positions. Four-channel tactile sensors are integrated into the joint sheaths of the robotic hand to measure the magnitude of pressure and the direction of the force, relative to the center of the sensor during object grasping.

**Figure 6:**
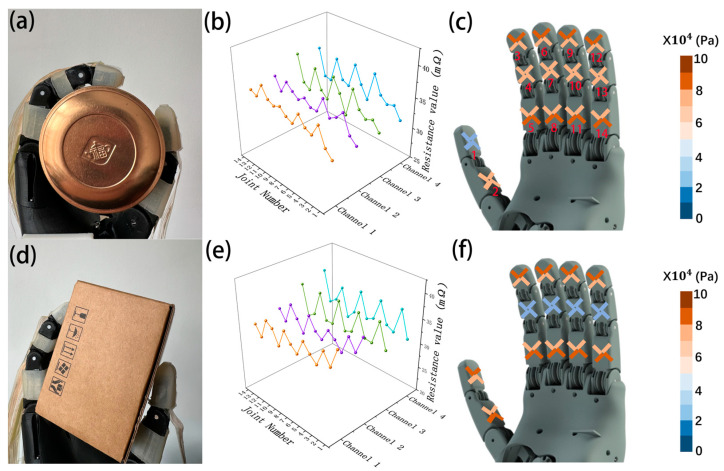
(**a**–**c**) respectively depict the physical object, experimental data, and force illustration of the underactuated robotic hand grasping a cylinder. (**d**–**f**) similarly represent the physical object, experimental data, and force illustration of the underactuated robotic hand grasping a cylinder.

(1) Robotic hand grasping cylindrical object:

Once the robotic hand securely grasps the object, the tactile sensors on the joints of the underactuated robotic hand’s index, middle, ring, and little fingers all make contact with the cylinder. Upon visual inspection, during the grasp of the cylinder, channels 1 and 2 on the tactile sensors of the distal phalanges of these four fingers experience significant pressure, while channels 3 and 4 are lightly pressed; the tactile sensors on the middle phalanges have evenly distributed pressure across all four channels; and on the proximal phalanges, channels 3 and 4 experience significant pressure, while channels 1 and 2 are lightly pressed. The tactile sensors on the proximal phalanx of the thumb experience evenly distributed pressure across all four channels, while the distal phalanx does not contact the object (as shown in Figure 6a).

(2) Robotic hand grasping rectangular object:

When grasping a rectangular prism, the tactile sensors on the distal and proximal phalanges of the under-actuated robotic hand’s index, middle, ring, and little fingers make contact with the prism in a manner consistent with grasping a cylinder. However, the tactile sensors on the middle phalanges of these fingers do not come into contact with the object. On the proximal phalanges of the thumb, channels 3 and 4 of the sensors experience significant force while, on the distal phalanges, channels 1 and 2 of the tactile sensors experience noticeable pressure (see Figure 6d).

The study collected 56 resistance value signals from the four-channel tactile sensors on a total of 14 joints of the robotic hand in the above two conditions. These sensors, proposed in this research, exhibit excellent stability. The mean values of each channel were calculated after stabilization, and are shown in Figure 6b,e. The variation in sensor resistance values on each joint corresponds to different stresses they experience, which match conclusions drawn from visual observations for underactuated robotic hand stress, as depicted in Figure 6c,f. Thus, the tactile sensor proposed in this study can effectively perceive the force exerted on robotic fingers, offering crucial guidance for future research on robotic hand grasping and object recognition through tactile feedback.

## 4. Discussion

This study conducts experiments on a single channel of a four-channel tactile sensor under the conditions of applied loads and repeated pressing at various room temperatures. The results not only match the theoretical model but also demonstrate the minimal temperature sensitivity (readout fluctuations less than 0.01%), the high stability (fluctuations less than 0.4%), the rapid response and recovery times (0.2 s and 0.15 s, respectively), and high linearity (R^2^ greater than 0.97) of the sensor. In addition, the experiments of applying stresses to different positions of the sensor verified the accuracy of the formulas for azimuth angle and normal resultant force through the data of the four channels, in which the discrepancies between the experimental and theoretical values are lower than 0.3% and 4%, respectively.

Compared to methods that use anisotropic structures, such as pyramids, hemispheres, and cylinders, [32] to enhance sensor sensitivity at the expense of linearity, the tactile sensor channel developed in this paper employs a 120° zigzag configuration for a single channel, which not only improves the response characteristics but also maximizes linearity. Furthermore, the micro-honeycomb electrodes (MHE)-based tactile sensor with a multi-touch mechanism [39] is technologically advanced but complicated to prepare, does not facilitate miniaturization and integration, and also has a higher cost. In contrast, the four-channel tactile sensor used in this study is made from Ecoflex^TM^00-30 and EGaIn alloy. It can be mass-produced and miniaturized by 3D-printed molds, simplifying the fabrication process and significantly reducing costs.

The four-channel tactile sensor developed in this study shows significant potential, for applications in several fields that require precise tactile feedback, because of its high sensitivity, stability, and rapid response. The sensor can accurately monitor touch and pressure in smart wearable devices, such as smartwatches and health-monitoring bracelets, in order to enhance the interaction experience of users. In medical prosthetics and service robots that interact with humans, it provides sensory feedback similar to that of a real limb and improves operational accuracy. In VR and AR systems, the quick response and stability of the sensor can deliver realistic tactile feedback and enrich the immersive experience. Additionally, it excels in safety monitoring and industrial automation, such as fall detection for the elderly and precision assembly, so as to ensure product quality and productivity. These characteristics indicate that the sensor has broad application prospects across various industries in the future.

## 5. Conclusions

The study presents a novel four-channel tactile sensor that uses a mathematical model to calculate the magnitude of normal force and the direction of point of action, relative to the sensor’s center, based on the resistance changes in the four channels. This sensor demonstrates high sensitivity and accuracy. Moreover, applying the sensor to the sheaths of underactuated robotic fingers can effectively solve the difficulty involved in identifying contact points of stretchable sensors during object grasping, thus significantly improving grasping precision and recognition capabilities. It is worth noting that EGaIn alloy is liquid at room temperature, and excessive load may cause leakage. Therefore, future application development should focus on improving the encapsulation techniques of EGaIn alloy.

## Figures and Tables

**Table 1:** Stress applied at various coordinates of the sensor and the resultant force in the four channels.

Location of Force	$R_{1}\;(m\Omega )$	$R_{2}\;(m\Omega )$	$R_{3}\;(m\Omega )$	$R_{4}\;(m\Omega )$	$F_{sum}\;(N)$	θ (°)
(0.6, 0.6)	38.13	38.34	36.07	36.28	3.41	44.77
(1.2, 1.2)	40.09	40.21	34.87	35.06	3.42	45.65
(1.8, 1.8)	42.12	42.23	31.75	31.68	3.47	44.73
(0, 0.6)	38.05	40.17	38.32	34.10	3.42	44.63
(0, 1.2)	37.34	41.23	37.89	32.21	3.48	45.51
(0, 1.8)	36.18	42.97	36.12	31.69	3.46	44.97
(0, 0)	37.45	36.56	37.12	37.16	3.38	45.03

## Data Availability

Data are contained within the article.
